# Inducible Prmt1 ablation in adult vascular smooth muscle leads to contractile dysfunction and aortic dissection

**DOI:** 10.1038/s12276-021-00684-x

**Published:** 2021-10-11

**Authors:** Jung-Hoon Pyun, Byeong-Yun Ahn, Tuan Anh Vuong, Su Woo Kim, Yunju Jo, Jaehyung Jeon, Seung Ho Baek, Jaewon Kim, Sungsu Park, Gyu-Un Bae, Jun-Hyuk Choi, Jae-Ryong Kim, Dongryeol Ryu, Sang-Jin Lee, Jong-Sun Kang

**Affiliations:** 1 Department of Molecular Cell Biology, Sungkyunkwan University School of Medicine, Suwon, South Korea; 2 Single Cell Network Research Center, Sungkyunkwan University School of Medicine, Suwon, South Korea; 3 Research Institute of Aging Related Diseases, AniMusCure, Inc, Suwon, South Korea; 4 School of Mechanical Engineering, Sungkyunkwan University, Suwon, South Korea; 5 Department of Global Biomedical Engineering, Sungkyunkwan University, Suwon, South Korea; 6 College of Pharmacy, Sookmyung Women’s University, Seoul, South Korea; 7 Department of Pathology, Yeungnam University College of Medicine, Daegu, South Korea; 8 Department of Biochemistry and Molecular Biology, Smart-aging Convergence Research Center, Yeungnam University College of Medicine, Daegu, South Korea

**Keywords:** Epigenetics, Cardiovascular diseases

## Abstract

Vascular smooth muscle cells (VSMCs) have remarkable plasticity in response to diverse environmental cues. Although these cells are versatile, chronic stress can trigger VSMC dysfunction, which ultimately leads to vascular diseases such as aortic aneurysm and atherosclerosis. Protein arginine methyltransferase 1 (Prmt1) is a major enzyme catalyzing asymmetric arginine dimethylation of proteins that are sources of asymmetric dimethylarginine (ADMA), an endogenous inhibitor of nitric oxide synthase. Although a potential role of Prmt1 in vascular pathogenesis has been proposed, its role in vascular function has yet to be clarified. Here, we investigated the role and underlying mechanism of Prmt1 in vascular smooth muscle contractility and function. The expression of PRMT1 and contractile-related genes was significantly decreased in the aortas of elderly humans and patients with aortic aneurysms. Mice with VSMC-specific Prmt1 ablation (smKO) exhibited partial lethality, low blood pressure and aortic dilation. The Prmt1-ablated aortas showed aortic dissection with elastic fiber degeneration and cell death. Ex vivo and in vitro analyses indicated that Prmt1 ablation significantly decreased the contractility of the aorta and traction forces of VSMCs. Prmt1 ablation downregulated the expression of contractile genes such as myocardin while upregulating the expression of synthetic genes, thus causing the contractile to synthetic phenotypic switch of VSMCs. In addition, mechanistic studies demonstrated that Prmt1 directly regulates myocardin gene activation by modulating epigenetic histone modifications in the myocardin promoter region. Thus, our study demonstrates that VSMC Prmt1 is essential for vascular homeostasis and that its ablation causes aortic dilation/dissection through impaired myocardin expression.

## Heart disease: A switch that helps hearts coordinate contraction

A protein that stabilizes the contractile function of cardiac muscle may act as an important safeguard against cardiovascular disorders. Several studies have hinted that the enzyme protein arginine methyltransferase 1 (Prmt1) may help preserve healthy cardiovascular function, and researchers led by Sang-Jin Lee at AniMusCure and Jong-Sun Kang of Sungkyukwan University in Suwon, South Korea, have produced evidence strongly supporting this idea. Mice lacking Prmt1 in their heart muscle exhibited clear signs of cardiac dysfunction including degeneration of the contractile fibers that coordinate beating. The researchers subsequently determined that the absence of Prmt1 causes muscle cells to switch to a type associated with damage repair and synthesis, which is ill-suited to routine heart function. Similar switching has been seen in various heart disorders, and treatments that boost Prmt1 activity could confer robust protection against these disorders.

## Introduction

The contractile property of vascular smooth muscle cells (VSMCs) is the key property of vascular functions^1^. Arterial stiffness and decreased contractile properties are highly associated with vascular aging and vascular diseases^2,3^. In response to injury, VSMCs begin to dedifferentiate, proliferate and migrate to damaged sites, adopting a synthetic phenotype linked with decreased contractile properties^1,4^. This phenotypic switch from contractile to synthetic is a major determinant of the development and progression of vascular diseases, such as aortic aneurysm/dissection and atherosclerosis^5–7^. Multiple mechanisms have been proposed to contribute to aortic aneurysm/dissection, including vascular inflammation, oxidative stress, extracellular matrix degradation, and VSMC dedifferentiation^8,9^. Several classes of genetic perturbations are directly linked with VSMC dysfunction. Mutations in contractile component genes (ACTA2, MYH11, MYLK) or components of transforming growth factor-beta signaling are implicated in VSMC dysfunction^10–13^. Furthermore, transcription factors such as serum response factor (SRF) and its interacting protein myocardin play essential roles in the development and differentiation of VSMCs^14–17^. Myocardin is expressed in developing and adult VSMCs and appears to be critical for the survival of VSMCs^18^. Myocardin-deficient mice died during early embryogenesis with a lack of differentiated smooth muscle cells^18,19^. Mice lacking myocardin in postnatal smooth muscle cells exhibited decreased expression of contractile genes and developed arterial aneurysms/dissection^18^.

Protein arginine methyltransferase 1 (Prmt1) catalyzes asymmetric arginine dimethylation of substrates and is the predominant Prmt in mammalian cells involved in the regulation of cell proliferation, differentiation, and survival. The dimethylation of histone H4 at arginine 3 (H4R3) by Prmt1 facilitates gene activation through the recruitment of several histone acetyltransferases, such as p300^20–22^. Recent studies have proposed a critical role of Prmt1 in cardiac function^23,24^. Cardiomyocyte-specific Prmt1 deletion in mice led to dilated cardiomyopathy, heart failure, and lethality within 2 months of age^23^. Prmt1 regulates alternative splicing transition occurring in the early postnatal days and Ca^2+^/calmodulin-dependent protein kinase II suppression is critical for the control of the contractile function of cardiomyocytes^24^. In the vascular system, Prmt1 deregulation is predicted to be associated with endothelial dysfunction and vascular diseases via the production of asymmetric dimethylarginine (ADMA), an endogenous inhibitor of nitric oxide synthase^25–28^. Thus, Prmt1 inhibition seems to be a good strategy to treat vascular diseases related to endothelial dysfunction. In contrast, a different study proposed the protective role of Prmt1 against arteriosclerotic calcification caused by vascular smooth muscle ablation of low-density lipoprotein receptor-related protein 6 in a diabetic mouse model^29^. Although the exact role and mechanism of Prmt1 in vascular pathophysiology is unknown, these studies predict an important role of Prmt1 in vascular smooth muscle function and diseases. Our initial study using rat vascular smooth muscle cells (RVSMCs) showed that Prmt1 activity is important for contractility and resistance to the mechanical stretch of VSMCs. Thus, we aimed to determine the in vivo role of Prmt1 in vascular smooth muscle function by generating tamoxifen (tmx)-inducible SM22α-Cre(ERT2);Prmt1(fl/fl) mouse model. Mice lacking VSMC-Prmt1 exhibited aortic dissection, vascular fibrosis, and hypotension concurrent with partial lethality. VSMC Prmt1 deficiency in mice resulted in impaired contractile properties accompanied by altered expression of genes related to the contractile to synthetic switch. Among the altered genes, Prmt1 inhibition was tightly linked with decreased expression of myocardin and its target contractile genes. Prmt1 depletion decreased active histone modification, including asymmetric dimethylation of H4R3 and acetylation of histone H3 lysine 9 (H3K9) in the myocardial promoter region, but enhanced suppressive histone H3 lysine 27 (H3K27) trimethylation. Therefore, we concluded that Prmt1 is required for the maintenance of vascular smooth muscle contractility through myocardin induction.

## Materials and methods

### Animal studies

Prmt1^fl/fl^ mice were maintained as previously described^30^. For *Prmt1* ablation in VSMCs, Prmt1^fl/fl^ mice were crossed with SM22α-Cre^ERT2^ mice, which express tamoxifen (tmx)-inducible Cre recombinase under the transcriptional control of the SM22α (Tagln) promoter. For ablation of the Prmt1 gene in VSMCs, 8 to 10-week-old mice were injected intraperitoneally with 100 mg/kg tmx for 4 days, and then, after injection for 2 weeks, mice were fed *ad libitum* with a tmx-supplemented diet (Envigo, TD130859, Madison, WI). The animal experiments in this study were approved by the Institutional Animal Care and Use Committee of Sungkyunkwan University School of Medicine (SUSM) and complied with the animal experiment guidelines of the SUSUM Ethics Committee.

### Human samples

Human carotid artery tissues (*n* = 6) were obtained from patients with atherosclerosis at Yeungnam University Hospital (Daegu, Republic of Korea) who consented to surgical excision from 1995 to 2012 with Internal Review Board approval (YUMC 2015-01-017).

### Cell culture experiments

Primary RVSMCs were isolated from fresh rat aortas as previously described^31^. Briefly, the aorta was removed from 6-week-old male Sprague-Dawley rats. The medial layer of the aorta was separated from the adventitia by elastase (Worthington Biochemical, LS002279) and collagenase (Worthington Biochemical, LS04177) treatment. To preserve smooth muscle cell characteristics, we carried out all experiments at passages 2–4. To knock down Prmt1 in primary RVSMCs, we used an adenoviral expression system^23,30^.

RVSMCs and A7R5 (ATCC, CRL-1444) cells were cultured in DMEM (Dulbecco’s modified Eagle’s medium) supplemented with 10% FBS (fetal bovine serum) and 1% antibiotics. For transfection experiments, cells were transfected using Lipofectamine 2000 (Invitrogen, 11668). For analysis of the inhibitory effects of Prmt1, isolated RVSMCs or A7R5 cells were exposed to 20 μM furamidine (Fura; Sigma-Aldrich, A7154) for 24 h. For depletion of Prmt1 in RVSMCs, control shRNA (shCon) or Prmt1 shRNA (shPrmt1) was expressed by using an adenoviral expression system, as previously described^32^.

### Protein analysis

Western blot analyses were carried out as previously described^33^. Briefly, cultured cells or homogenized tissue samples were lysed in RIPA buffer (protease inhibitor cocktail (Roche, 1183617001), pH 8.0; 150 mmol/L NaCl; 1 mmol/L EDTA; 1% Triton X-100; 10 mml/L Tris-HCl). The primary antibodies used in this study are listed in Supplementary Table 1.

### RNA analysis

Total RNA was extracted from aortic tissue samples and cultured cells using TRIzol (Invitrogen, 15596026). cDNA was amplified using a PrimeScript RT reagent kit (TaKaRa, RR064A) according to the manufacturer’s instructions. SYBR Premix Ex Taq (TaKaRa, RR420) was used to analyze gene expression on a Thermal Cycler Dice Real-Time System (TaKaRa, TP950) according to the manufacturer’s instructions. The primer sequences used in this study are listed in Supplementary Table 2. RNA sequencing was performed with an Agilent 2100 bioanalyzer using an RNA 6000 Nano Chip system (Agilent Technologies). RNA sequencing data were analyzed by using ExDEGAv1.61 (e-Biogen). For analysis of functional gene expression, Gene Set Enrichment Analysis (GSEA; http://www.broadinstitute.org/gsea) was performed as described previously (PMID: 32096917)^33^. All GSEA plots, including GSEA enrichment plots, were generated with GSEA software (Broad Institute; https://www.gsea-msigdb.org/gsea/). The enrichment map was visualized with Cytoscape (V3.8.2; https://cytoscape.org). The transcriptomes of the human artery were obtained from the portal of the Genotype-Tissue Expression (GTEx) project and visualized with the RStudio and R packages ggplot2.

### Stretching device for uniaxial stretching

The stretching device was composed of a stretcher (26 × 49 × 10 mm, W × L × H) and a disposable cell seeding chamber (11 × 21 × 6 mm, W × L × H). The stretcher was completed by assembling 4 functional parts: pneumatic piston (Misumi, Tokyo, Japan), linear shafts (Misumi, Tokyo, Japan), and tubing (Nam Kang K-flex, Yongin, Korea). The holder was made by aluminum manufacturing (Metrotech, Gunpo, Korea). The cell seeding chamber was made of polydimethylsiloxane (PDMS) (Sylgard 184 silicone elastomer: Dow Corning, MI, USA) using an aluminium mold (Metrotech, Gunpo, Korea). The mixture of PDMS prepolymer and its curing agent at a 10:1 (w/w) ratio was cured in an aluminium mold at 80 °C for 60 min and peeled from the mold. A thin layer of PDMS membrane at 200 ${\upmu}$m was prepared by spin-coating the PDMS mixture on a 4-inch silicon wafer for 30 s at 450 rpm using a spin coater (Spin-1200D; MIDAS, Daejeon, Korea) and curing at 80 °C for 60 min. The cured membrane was cut into a piece (21 × 11 mm; W × L) using a knife. The cut was treated with O_2_ plasma for 30 s and bonded to the bottom of the chamber^34^.

### Elastic pillars

Pillars (900 nm in diameter, 2 µm in height, and 1.8 μm in center-to-center distance between pillars) were fabricated using soft lithography^35^. First, the mold for the pillars was fabricated by standard photolithography with reactive ion etching. The mold had an array of holes on a silicon wafer. The mold was silanized as described before. The PDMS mixture was prepared as described above and spin-coated onto the mold at 500 rpm for 10 s with an acceleration of 100 rpm/s, followed by spin coating at 1800 rpm for 30 s with an acceleration of 300 rpm/s. Next, the spin-coated mold with PDMS was degassed and cured at 80 °C for 4 h and 30 min until Young’s modulus of PDMS reached 2 MPa. Finally, the cured pillar array was carefully peeled off from the mold.

The bending stiffness of the pillar was calculated by the Euler-Bernoulli beam theory:

$${{{\mathrm{k}}}} = \frac{3}{{64}}\pi {{{\mathrm{E}}}}\frac{{{{{\mathrm{D}}}}^4}}{{{{{\mathrm{L}}}}^3}}$$

where D is the diameter, L is the length, and E is the Young’s modulus of the pillar. The bending stiffness (k) of the pillar arrays used in this study was 24.15 nN/µm.

Images of A7R5 RVSMCs on elastic pillars were captured using a live cell chamber at 1 Hz under a fluorescence microscope (Deltavision, GE Healthcare, Chicago, IL, USA) equipped with a camera (CoolSNAP HQ2, Photometrics) at 37 °C and 5% humidity. The deflection of each pillar in the images was analyzed using the pillar tracking plugin (PillarTracker 1.1.6 version) of ImageJ software, which uses the pillar reconstruction algorithm to set up an exact grid of the pillar arrays^35^. PillarTracker was used to create a deflection map, and it could also automatically detect the position of pillars and their deflection value. To remove displacement caused by stage drift, we subtracted the average displacement of the reference pillars with no cell contact from the displacement data of pillars deflected by A7R5 RVSMCs.

### Histology and immunostaining

For the histological analysis of aortic tissue, aortas were removed from PBS-perfused mice, fixed with 4% paraformaldehyde (PFA), embedded in paraffin, and sectioned at 6 μm thickness. Slides were stained with hematoxylin and eosin (H&E; BBC, Biochemical), Masson’s trichrome (MT; Abcam, ab150686), and Verhoeff-Van Gieson (VVG). For the immunohistochemical examination, deparaffinized samples were boiled in Tris-EDTA buffer (pH 9.0, 0.05% Tween-20) for antigen retrieval, followed by a standard protocol for immunostaining. For analysis of cell death, aortic sections were processed by using a Click-iT TUNEL assay imaging kit (Invitrogen, C10243). Immunocytochemistry was performed as previously described^36^. In brief, cells cultured on coverslips were fixed using 4% PFA and permeabilized with 0.1% Triton X-100 in PBS. Cells were then blocked with 10% goat serum diluted in PBS. The cells were incubated with primary antibodies diluted in the blocking solution overnight at 4 °C. The cells were incubated with Alexa 488- or Alexa 568-conjugated secondary antibodies (Invitrogen) for 1 h at room temperature (RT). The stained cells were mounted with Mowiol solution. Fluorescent images were obtained using an LSM-710 confocal microscope system (Carl Zeiss) and ZEN software (Carl Zeiss).

### Chromatin immunoprecipitation (ChIP) assay

ChIP assays were carried out as previously described^37^. In brief, A7R5 cells infected with adenoviral control or shPrmt1 were fixed using 1% formaldehyde for 10 min at RT and quenched with 125 mM glycine for 5 min at RT. After lysis of the harvested cells, lysates were sonicated to shear chromatin to a length of 200–1000 bp. Lysates containing fragmented chromatin were incubated with antibodies overnight at 4 °C and precipitated with Protein A-Agarose/salmon sperm DNA (Millipore, 16-157) according to the manufacturer’s protocol. Eluted chromatin from beads was extracted using phenol-chloroform:isoamyl alcohol (Sigma, 77617) and processed through ethanol precipitation. The antibodies and PCR primer sequences used in this assay are presented in Supplementary Tables 1 and 2.

### Blood pressure measurements and echocardiograms

The systolic blood pressure of the mice was measured with a noninvasive blood pressure system (AD instrument, IN125/M). Briefly, mice were trained for blood pressure analysis on a daily basis for 2 weeks. After the adjustment period, blood pressure was measured once a week.

Echocardiography analysis was carried out as previously described^38^. In brief, echocardiography was performed as an M-mode image derived from the short axis of the lateral ventricle (LV) using a Vevo LAZR-X VisualSonics machine (BIORP of Korea Basic Science Institute) with a 40 MHz probe (VisualSonics). Mice were anesthetized in an induction chamber using 2–3% isoflurane and then transferred to a heated electrocardiography platform for heart rate monitoring. For measurement of aortic arch diameter, B-mode images of aortic arcs were also taken. Data analysis was performed using the Vevo LAB 3.1 cardiac analysis package. Each analysis was performed by the investigator in a blinded trial concerning the genotype.

### Wire myography

For isometric tension measurement, thoracic aortas were isolated from mice and rinsed in Krebs solution. After careful removal of connective tissue, the aorta was cut into 2-mm-long segments and mounted in a wire myography chamber (Danish Myo Technology, 620 M) filled with aerated (95% O_2_-5% CO_2_) Krebs solution and maintained at 37 °C. For equilibration, isolated aortic rings were stretched until receiving resting tension and exposed to 60 mM KCl Krebs solution to analyze contractility. For analysis of the contractile response to cumulatively administered vasoconstrictive agonists, each aorta was treated with PE (Sigma, P6126). Assessment of aorta tension was performed in a blinded trial.

### Serum ADMA and nitric oxide (NO) measurement

Serum samples isolated from mice were used for measuring ADMA (MyBioSource; MBS263225) and NO (Invitrogen, EMSNO) according to the manufacturers’ instructions.

### Statistics

Statistical comparisons of the results were analyzed with two-tailed Student’s *t*-tests when two cases were compared. When data did not meet the normality criteria, Mann–Whitney rank-sum tests were performed instead, as indicated in the figure legends. Statistical analysis for more than three groups or factors was conducted using analysis of variance (ANOVA) followed by a post hoc test. The errors of the pillar deflections were corrected by subtracting the reference pillar deflection. A *P* value<0.05 was considered significant.

## Results

### Prmt1 is expressed in aortic VSMCs and decreases in aged or diseased aortas

**Fig. 1:**
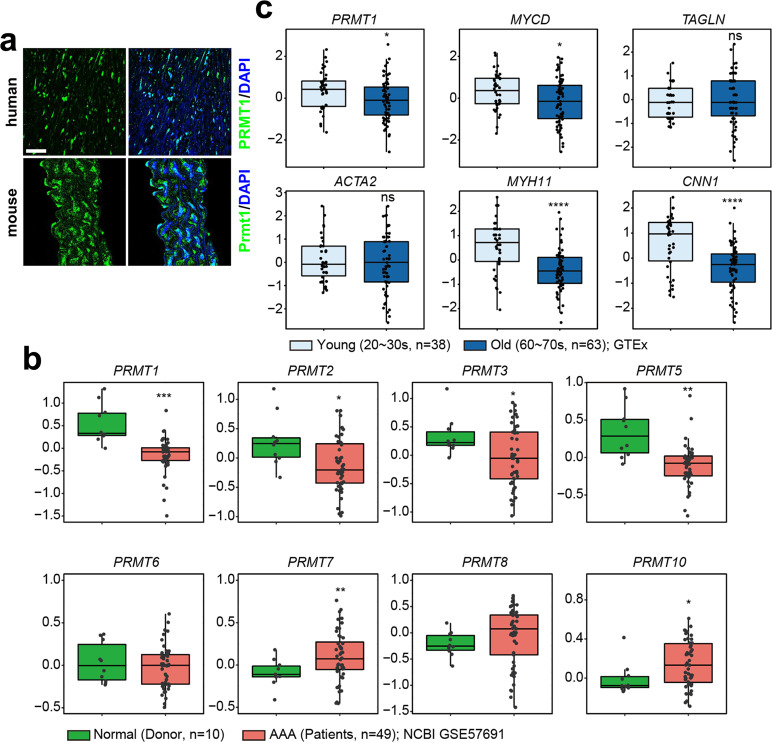
Prmt1 is expressed in aortic VSMCs and is decreased in aged or diseased human aortas. **a** Immunostaining images for PRMT1 in VSMCs of the human artery and mouse aorta. Scale bar: 40 μm. **b** Boxplots showing the expression of *PRMTs* in aortic specimens from either healthy donors (*n* = 10, green) or patients with abdominal aortic aneurysm (AAA, *n* = 49, red) (GSE57691). **c** Comparison of the expression of *PRMT1*, *Myocardin* (*MYCD*), and contractile genes in aortas from either the young (20–30 s, *n* = 38, light blue) or the old (60–70 s, *n* = 63, blue) group of human aortas (GTEx). Statistical significance was determined with two-tailed Student’s *t*-tests (**b**) or Mann–Whitney U tests (**c**). *P < 0.05; **P < 0.01**;** ***P < 0.001; ****P < 0.0001; n.s. not significant.

To investigate the role of Prmt1 in vascular function, we first characterized the expression of Prmt1 in human and mouse aortas. Immunostaining data showed that Prmt1 was highly expressed in the medial smooth muscle region of the aorta (Fig. 1a). Next, we analyzed the datasets (GSE57691)^39^ obtained from aortic specimens of either healthy donors or patients with abdominal aortic aneurysm (AAA) for the expression of PRMTs. PRMT1 and PRMT5 levels were significantly downregulated, while PRMT7 and PRMT10 levels were substantially increased, suggesting the potential involvement of these PRMTs in vascular function (Fig. 1b). We then compared the expression of PRMT1 and contractile program genes (MYOCARDIN, TAGLN, ACTA2, MYH11, CNN1) in datasets from either young (20–30 s) or old (60–70 s) groups of human artery aortas^40^ (Fig. 1c). The results revealed that the expression of PRMT1, MYOCARDIN, MYH11, and CNN1 was significantly reduced in the old aortic samples. These data suggest that Prmt1 might play a key role in contractile vascular function.

### Inducible ablation of Prmt1 in adult vascular smooth muscle causes lethality, hypotension and aortic dilation

**Fig. 2:**
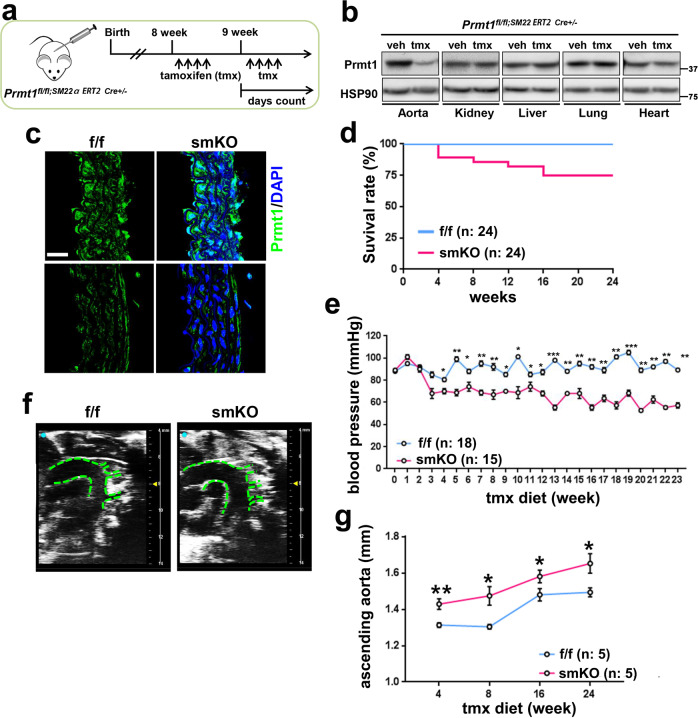
Inducible ablation of Prmt1 in adult vascular smooth muscle causes lethality, hypotension, and aortic dilation. **a** Experimental scheme for generating smooth muscle-specific Prmt1-depleted mice. The Prmt1^*fl/fl*;Sm22α-Cre*ERT2*^ mice were treated with tmx by intraperitoneal injections as indicated and fed tmx chow thereafter to maintain Prmt1 ablation. **b** Protein analysis of Prmt1 expression in the aorta, kidney, liver, lung and heart isolated from the control f/f- and tmx-treated smKO mice. **c** Immunohistochemistry images of the f/f and smKO aortic sections. Scale bar: 40 μm. **d** Survival curve for the f/f (*n* = 24) and smKO (*n* = 24) mice over 24 weeks. **e** Systolic blood pressure of the f/f (*n* = 18) and smKO (*n* = 15) mice. Data represent the mean ± SEM. *P < 0.05, **P < 0.01, ***P < 0.001, Student’s *t*-test. **f** Representative ultrasound images of the ascending aorta in the f/f- and 10-week-tmx-treated smKO mice. **g** Quantification of the ascending aortic dimensions of the f/f (*n* = 5) and smKO (*n* = 5) mice. Data represent the mean ± SD. *P < 0.05*,* **P < 0.01, Student’s *t-*test.

To examine the in vivo role of Prmt1 in vascular function, we generated mice harboring a tmx-inducible, SM22α-specific null mutation in the Prmt1 gene (Prmt1^(fl/fl)/SM22α-CreERT2^), and smooth muscle-specific deletion of Prmt1 (smKO) was carried out as described in the scheme by tmx treatment (Fig. 2a). Prmt1 levels were greatly decreased in the aorta of the smKO mice, while other tissues of the smKO mice did not exhibit clear differences in Prmt1 levels compared with the control f/f tissues (Fig. 2b). By immunostaining, we confirmed that Prmt1 expression was greatly reduced in smooth muscle cells of the aortas (Fig. 2c).

Next, we examined the phenotype of the smKO mice in detail. The smKO mice did not exhibit any overt alteration in body weight or weights of organs such as kidney, lung, and hearts compared to the f/f mice (Supplementary Fig. 1a–d). Starting 3 months after Prmt1 ablation, the lethality of the smKO mice was observed, and approximately 25% of the mice died within 4 months of Prmt1 ablation without any additional insult (Fig. 2d). Starting 3 weeks post-tmx treatment (3 weeks-tmx), the smKO mice exhibited lower systolic blood pressure than the f/f mice (Fig. 2e). Echocardiogram analysis revealed dilated ascending aortas in the smKO mice compared to the f/f mice (Fig. 2f, g). The serum ADMA level in the smKO mice was reduced, while the NO level was not significantly changed in these mice, implying that the phenotype of the smKO mice might not be associated with the prominent effects of ADMA on the vascular system (Supplementary Fig. 1e, f). Taken together, these results suggest that Prmt1 depletion in vascular smooth muscle causes lethality and exacerbates vascular dysfunction.

### Prmt1 deficiency leads to VSMC apoptosis and medial degeneration

**Fig. 3:**
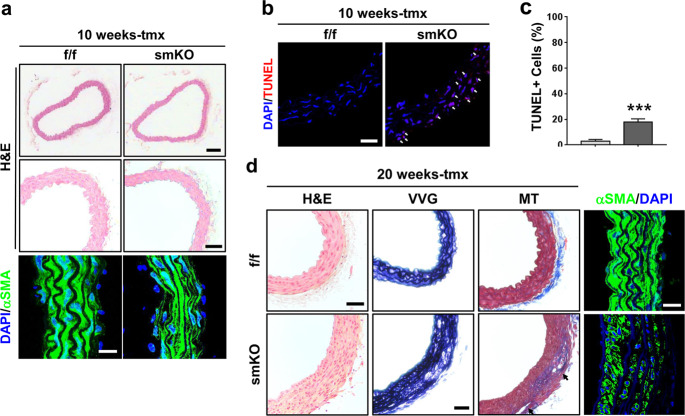
Prmt1-deficient aortas exhibit VSMC apoptosis and medial degeneration. **a** Histological analysis of thoracic aortas isolated from the f/f and smKO mice treated with tmx for 10 weeks. Representative images of hematoxylin and eosin (H&E) staining. Scale bar: 100 μm (upper), scale bar: 50 μm (bottom). Immunostaining for αSMA on the f/f and smKO aortas. Scale bar: 40 μm. **b** Representative confocal microscopic images of TUNEL-positive nuclei (red) in the f/f and smKO mouse aortas. Scale bar: 50 μm. **c** Quantification of TUNEL-positive nuclei in Panel **b**. Data represent the mean ± SD. ***P < 0.01, Student’s *t*-test. **d** Histological analysis of thoracic aortas isolated from the f/f and smKO mice treated with tmx for 20 weeks. Representative images of H&E, Verhoeff-Van Gieson (VVG), and Masson’s trichrome (MT) staining. Scale bar: 100 μm. Immunostaining for αSMA in the f/f and smKO aortas. Scale bar: 40 μm.

Histological analysis of the f/f and smKO aortas after 10 weeks of tmx treatment revealed structural disorganization of smooth muscle layers in the smKO aortas (Fig. 3a). Consistent with the dilated aortas in the smKO mice, the expression of alpha-smooth muscle actin (αSMA) was greatly reduced in the smKO aortas compared to the f/f aortas. We assessed pathological changes in the thoracic aorta by Prmt1 ablation. TUNEL staining revealed that VSCMs in the thoracic aorta of the smKO mice at 10 weeks of tmx treatment exhibited massive cell death of approximately 22%, in contrast to the very rare TUNEL-positive cells in the f/f aorta (Fig. 3b, c). Histological analysis of the f/f and smKO aortas after 20 weeks of tmx treatment revealed structural disorganization of smooth muscle layers in the smKO aortas (Fig. 3d). VVG and MT staining and αSMA immunostaining showed media degeneration, including elastic fiber fragmentation and collagen accumulation, in the smKO aortas. Furthermore, aortic dissection lesions were observed in the smKO aortas. These findings suggest a major role of Prmt1 in vascular homeostasis. Taken together, these data suggest that Prmt1 is required for the maintenance of VSMC survival and vascular homeostasis.

### Prmt1 deficiency or inhibition reduces VSMC contractility

**Fig. 4:**
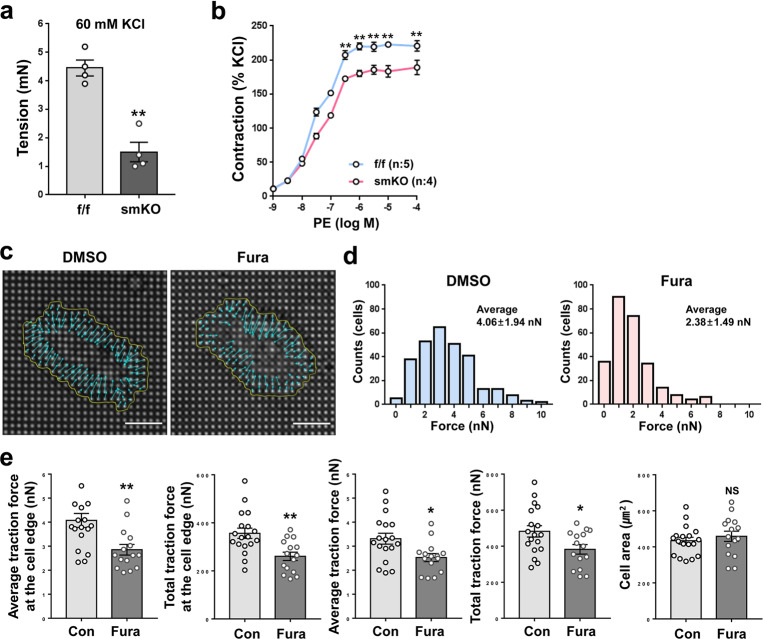
Prmt1-deficient aortas exhibit decreased contractility, and Prmt1 inhibition reduces the traction force of VSMCs. **a** Analysis of the elasticity of aortic rings from the f/f and smKO mice as measured by wire myography. Data represent the mean ± SEM. **P < 0.01, Student’s *t*-test. **b** Analysis of the contraction force of aortic rings isolated from the f/f and smKO mice in the presence of increasing concentrations of phenylephrine. Data represent the mean ± SEM. **P < 0.01, Student’s *t*-test. **c** Representative images of the pillar deflections and magnified pillar deflections at the edge of the DMSO- or Fura-treated RVMSCs (*n* = 15–18). **d** The force range of the DSMO- or Fura-treated RVSMCs. **e** Average traction forc**e** and total traction force of pillars under the DMSO- or Fura-treated cells. Data represent the mean ± SD. *P < 0.05, **P < 0.01, Student’s *t*-test.

Next, we assessed the contractile strength of the f/f and smKO thoracic aortas at 20 weeks of tmx treatment by using wire myography. Compared to aortas with a tension of 4.2 mN in the f/f group, the smKO aortas exhibited reduced tension of approximately 1.3 mN in response to depolarization with 60 mM KCl (Fig. 4a). The induction of contraction by treatment with different concentrations of phenylephrine (PE) post-KCl-mediated depolarization revealed the reduced contractile force of the smKO aortas compared to the f/f aortas (Fig. 4b). To investigate the role of Prmt1 activity on VSMC contractility, we treated RVSMCs with the vehicle DMSO or the Prmt1-specific inhibitor Fura for 24 h and plated the cells onto elastic pillars coated with Matrigel, followed by measurement of traction force. Fura treatment significantly reduced traction forces in RVSMCs compared to the control treatment (Fig. 4c–e). These data suggest a critical role of Prmt1 in the contractility of VSMCs.

### Prmt1 inhibition in VSMCs elevates cellular stress triggered by mechanical stretch

**Fig. 5:**
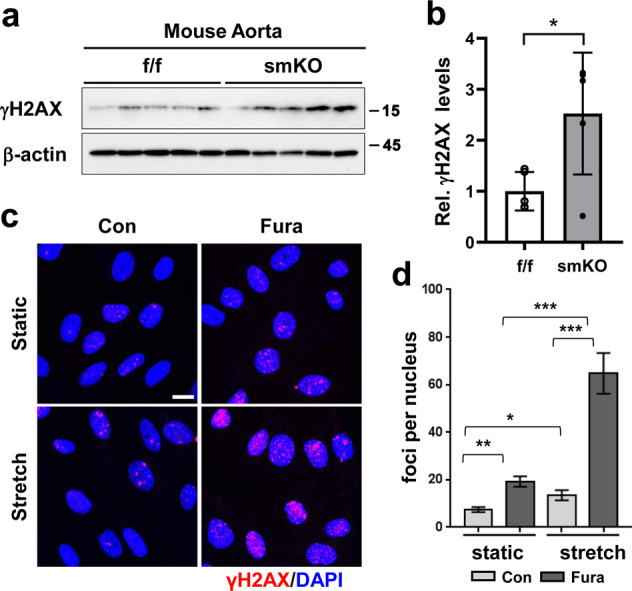
Prmt1 inhibition in VSMCs elevates cellular stress triggered by mechanical stretch. **a** Immunoblot analysis of γH2AX in the f/f and smKO aortas. **b** Quantification of the γH2AX intensity relative to β-actin. (*n* = 5). Data represent the mean ± SD. *P < 0.05, Student’s *t*-test. **c** Immunostaining for γH2AX in A7R5 cells treated with Fura followed by 10% cyclic stretch for 2 h. Scale bar: 50 μm. **d** Quantification of γH2AX-positive cells (red) (*n* = 89–114). Data represent the mean ± SD. *P < 0.05, **P < 0.01, ***P < 0.001, Student’s *t*-test.

For analysis of cellular stress in the Prmt1-ablated VSMCs, the f/f and smKO thoracic aortas were subjected to immunoblotting analysis for gamma-H2A histone family member X (γH2AX). The smKO aortas exhibited a significant increase in γH2AX levels compared to the f/f aortas (Fig. 5a, b). To assess the role of Prmt1 in the response to mechanical stress in RVSMCs, we utilized uniaxial cyclic stretching (10 to 15%, 0.67 Hz) to mimic in vivo mechanical stress. The control or Fura-treated RVSMCs were subjected to uniaxial cycling stretching and then immunostained for γH2AX to assess cellular stress. Mechanical stretching significantly increased the number of γH2AX foci in the control RVSMCs compared to that in the static condition (Fig. 5c, d). In contrast, Prmt1 inhibition augmented the formation of γH2AX foci under both static and mechanical stretch conditions; however, mechanical stretching greatly enhanced cellular stress in the Prmt1-inhibited cells. These results suggest that Prmt1 is important to resist mechanical stretch-triggered cellular stress in VSMCs.

### VSMC Prmt1 ablation results in the alteration of genes related to arterial diseases, abnormal glucose/cholesterol metabolism and abnormal heart morphology

**Fig. 6:**
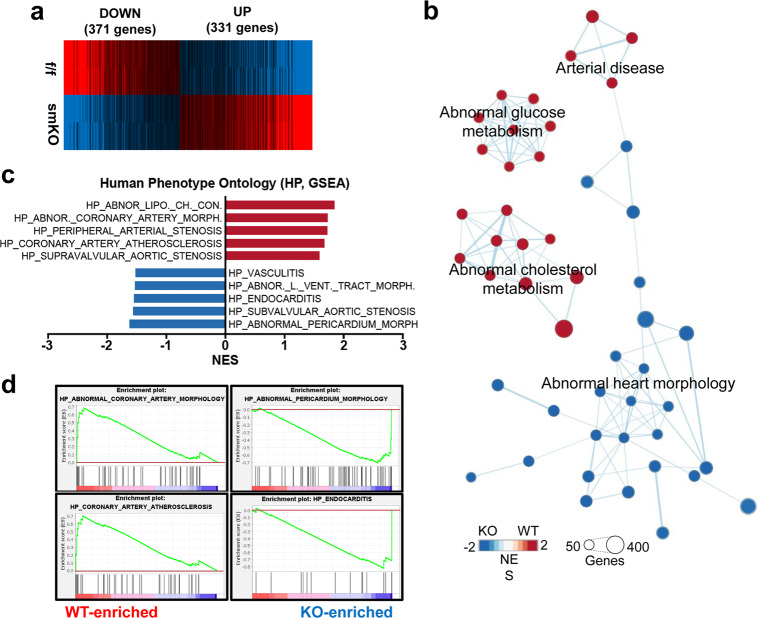
Gene expression profiles of aortas of the f/f and smKO mice. **a** Heatmap of reactome database clusters demonstrating the expression pattern of genes with up- and downregulated expression between the f/f and smKO aortic samples. **b** The enrichment map highlighting the results of Gene Set Enrichment Analysis (GSEA) of RNA samples from the f/f and smKO aortas (*n* = 2). **c** Bar charts presenting the normalized enrichment score (NES) of The Human Phenotype Ontology data (Molecular Signatures Database v7.2, GSEA, Broad Institute). **d** GSEA enrichment plots showing representative gene sets of The Human Phenotype Ontology database.

To determine the mechanisms underlying smKO pathology, we performed RNA sequencing analysis using aortas from the f/f and smKO mice after 2 weeks of tmx treatment when the blood pressure of the smKO animals was comparable to that of the f/f animals. The data revealed that 371 genes and 331 genes had significantly down- and upregulated expression, respectively (Fig. 6a). Gene set enrichment analysis revealed that genes related to abnormal heart morphology showed downregulated expression in the smKO aortas, while genes related to arterial diseases, abnormal glucose metabolism, and abnormal cholesterol metabolism showed significantly upregulated expression relative to those in the f/f aortas (Fig. 6b). Furthermore, the analysis of The Human Phenotype Ontology-related gene expression dataset revealed that the smKO aortas had significantly altered gene expression related to abnormal coronary artery morphology, peripheral arterial stenosis, coronary artery atherosclerosis, and supravalvular aortic stenosis (Fig. 6c, d). These data suggest that the phenotype observed in the smKO group resembles human arterial dysfunction-related pathologies.

### VSMC Prmt1 ablation decreases myocardial and contractile gene expression concurrent with upregulated expression of synthetic genes

**Fig. 7:**
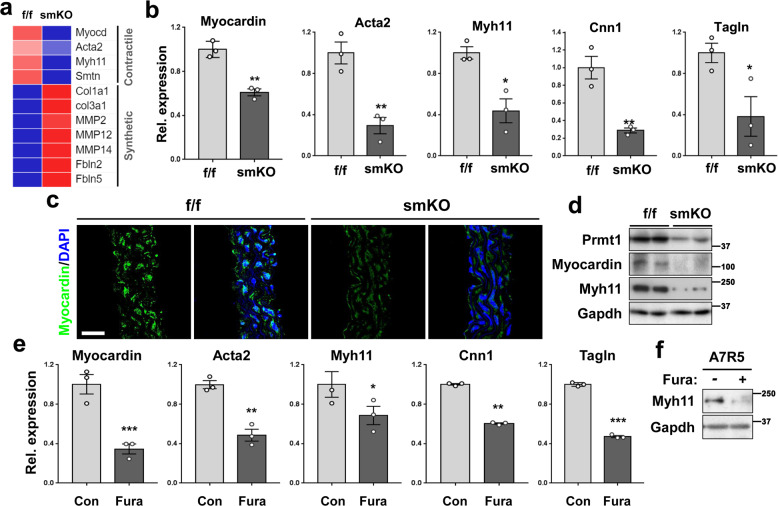
VSMC-Prmt1 ablation triggers alterations in the expression of genes related to the switch of contractile to synthetic phenotypes. **a** Expression pattern of contractile and synthetic markers in aortic tissue from the f/f and smKO mice (*n* = 2). **b** Relative RNA expression level of myocardin and its target genes in aortic tissue isolated from f/f or 10-week tmx-treated smKO mice (*n* = 3). Data represent the mean ± SEM. *P < 0.05, **P < 0.01, Student’s *t*-test. **c** Immunostaining images for myocardin (green) in thoracic aorta sections from the f/f or smKO mice. Scale bar: 40 μm. **d** Protein analysis of the f/f and smKO aortic tissues. **e** qRT-PCR analysis of myocardin, Acta2, Myh11, Cnn1, and Tagln in the A7R5 cells treated with Fura for 24 h. Data represent the mean ± SEM. *n* = 3. *P < 0.05, **P < 0.01, ***P < 0.001, Student’s *t*-test. **f** Immunoblot analysis of Myh11 in the A7R5 cells treated with Fura for 24 h.

In the close examination of RNA sequencing data, the smKO aortas exhibited decreased expression of myocardin and its target contractile genes (Acta2, Myh11, Smtn), while the expression of synthetic genes (Col1a1, Col3a1, MMP2, MMP14, Fbln2, Fbln5) was elevated (Fig. 7a). Quantitative RT-PCR analysis confirmed that the transcriptional levels of myocardin and its target contractile genes Acta2, Myh11, Cnn1, and Tagln were significantly reduced in the smKO aortas relative to the f/f aortas (Fig. 7b). Furthermore, the smKO aortas displayed greatly reduced myocardin immunostaining relative to the f/f aortas (Fig. 7c). Reduced protein levels of myocardin and Myh11 were also observed in the smKO aortas relative to the f/f aortas (Fig. 7d). In addition, Prmt1-mediated asymmetric dimethyl H4R3 (ASYM-H4R3), which is a marker for gene activation, was significantly reduced in the smKO aortas, regardless of the Prmt4 increase. However, the smKO aortas displayed a modest increase in Prmt5-mediated symmetric dimethyl H4R3 (SYM-H4R3), which is the marker for gene repression, with no alteration in Prmt5 levels (Supplementary Fig. 2a). These data imply that Prmt1 deletion in VSMCs independently dysregulated gene expression involving contractile gene expression. Similar to the data obtained from the aortic samples, the Prmt1-depleted primary RVSMCs and A7R5 cells showed decreased expression of myocardin and target contractile genes (Supplementary Fig. 2b, c). To further explore the relationship between Prmt1 and contractile genes, we examined 18 sets (*n* = 196–256) of VSMC transcriptomes from a public database (Supplementary Fig. 3). We found a positive correlation between Prmt1 and contractile genes, supporting the role of Prmt1 in the regulation of the contractile gene program. To test whether the methyltransferase activity of Prmt1 is required for the expression of myocardin and its target genes, we treated A7R5 cells with vehicle or Fura for 24 h and examined the expression of myocardin and its target genes by qRT-PCR (Fig. 7e). The expression level of Myh11 proteins was reduced in the Fura-treated A7R5 cells compared to the vehicle-treated cells (Fig. 7f). Similar to Prmt1 depletion, Prmt1 inhibition by Fura treatment also significantly reduced the expression of myocardin and its target genes, suggesting that the activity of Prmt1 is required for myocardin expression in VSMCs.

### Prmt1 depletion causes alteration in histone modification in the promoter region of the myocardin gene

**Fig. 8:**
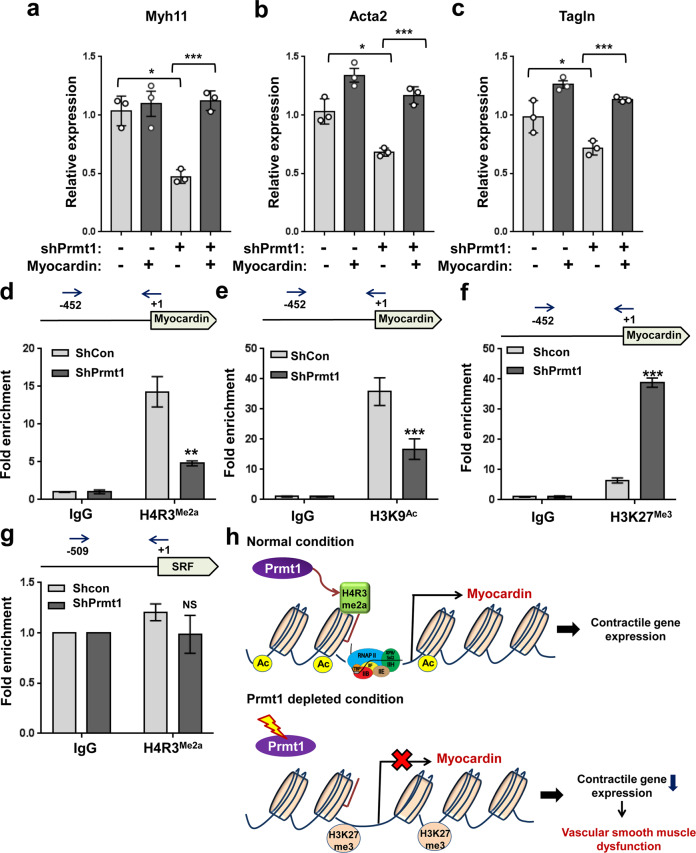
Prmt1 depletion alters active and repressive histone markers in the promoter region of myocardin. **a**–**c** Relative RNA expression levels of the contractile genes Myh11 (**a**), Acta2 (**b**) and Tagln (**c**) in the RVSMCs expressing a combination of shCon or shPrmt1 and myocardin. *n* = 3. Data represent the mean ± SEM. *P < 0.05, ***P < 0.001, Student’s *t*-test. **d**–**g** Chromatin immunoprecipitation (ChIP) assays of the shCon- or shPrmt1-expressing A7R5 cells for histone modification markers in the promoter regions of myocardin (+1 ~ -452) and SRF ( + 1 to −509) genes. Values are expressed as a percentile of input. *n* = 3. Data represent the mean ± SEM. NS: not significant, **P < 0.01, ***P < 0.001. Student’s *t*-test. **h** Working model summarizing the regulatory mechanism of myocardin expression and maintenance of vascular smooth muscle function by Prmt1-mediated histone modification.

Next, we examined whether myocardin expression can restore the expression of contractile genes in the Prmt1-depleted A7R5 cells. The reduction in Myh11, Acta2, and Tagln expression induced by Prmt1 deletion in A7R5 cells was attenuated by myocardin expression (Fig. 8a–c), suggesting that Prmt1 regulates contractile genes through myocardin. To gain insight into the mechanism of myocardin gene regulation by Prmt1, we examined epigenetic modifications of the myocardin promoter region. We first performed a ChIP assay with Prmt1 antibodies, but unfortunately, no commercially available antibodies were specific for ChIP. Thus, we detected Prmt1-mediated asymmetric dimethyl-H4R3 (H4R3^me2a^) in the myocardin promoter region (Fig. 8d–g). H4R3^me2a^ has strongly enriched at the myocardin promoter region in the control A7R5 cells, while Prmt1 depletion attenuated this enrichment. Consistently, acetylated H3K9 (H3K9^Ac^), another active histone marker, was also significantly reduced in the Prmt1-depleted cells. In contrast, repressive trimethyl-H3K27 (H3K27^me3^) enrichment was greatly enhanced in the Prmt1-depleted cells relative to the control cells. Interestingly, the promoter region of SRF did not show any accumulation of H4R3^me2a^ compared to the control IgG. This finding is consistent with the RNA sequencing data showing no alteration in the expression of SRF in the smKO aortas. Thus, we concluded that Prmt1 is required for the maintenance of vascular smooth muscle contractility by regulating myocardin expression.

## Discussion

In this study, we demonstrate the importance of Prmt1 in VSMC function to ensure vascular homeostasis. This is the first report to show the functional significance of protein arginine methylation in the vascular system. In the current study, we aimed to determine the functional importance of Prmt1 in vascular muscles by using inducible smooth muscle-specific Prmt1 knockout mice. Our data suggest that adequate Prmt1 activity is required to maintain vascular function. Loss of the contractile VSMC phenotype is associated with many vascular diseases, such as atherosclerosis and aortic aneurysm/dissection^7,41–43^. Ex vivo and in vitro analyses indicated that Prmt1 inhibition by gene depletion or Fura treatment significantly decreased the contractility of aortas and the traction forces of VSMCs.

Transcriptome analysis revealed a shift from the contractile to the synthetic gene program, and the levels of myocardin and its target genes were greatly decreased in the smKO aortas compared to the f/f aortas. Prmt1 might regulate the expression of myocardin through asymmetric dimethyl-H4R3 modification, which in turn modulates active and suppressive histone modification. Since no alteration in asymmetric dimethyl-H4R3 in the promoter region of the SRF gene was observed in the smKO aortas, this epigenetic regulation seems to be specific to the myocardin gene. Thus, VSMC Prmt1 deficiency attenuates myocardin expression, thereby inducing the phenotypic switch from contractile to synthetic, contributing to vascular dysfunction. This notion is further supported by the similar phenotypes of mice with ablated smooth muscle myocardin and smKO^18^. The genetic deletion of smooth muscle myocardin in mice led to lethality and aortic dissection. These phenotypes are also accompanied by the loss of the contractile phenotype coinciding with the activation of endoplasmic reticulum (ER) stress, autophagy and cell death^18^. Similarly, Prmt1 ablation also caused cell death of smooth muscles in aortas, likely contributing to aortic dilation. In the current study, we did not assess ER stress or autophagy in Prmt1-ablated smooth muscles in detail. Our previous studies on the role of Prmt1 in cardiac and skeletal muscles suggested that Prmt1 might also be critical to modulate ER stress and autophagy^30,37^. Ablation of Prmt1 in cardiomyocytes triggers ER stress and the unfolded protein response, likely contributing to cell death^42,44^. Furthermore, Prmt1-deficient skeletal muscle exhibited a dysregulated autophagic response leading to muscle wasting^30^. It is conceivable that Prmt1 ablation results in ER stress and unfolded protein responses through decreased myocardin expression associated with cardiovascular diseases.

To date, Prmt1 has been suggested to be a potential player in vascular pathogenesis and has emerged as a potential therapeutic target for vascular diseases^29,45^. This finding is based on the fact that Prmt1 is the major enzyme catalyzing asymmetric protein demethylation, the source of ADMA through protein degradation^26^. This pathological role of Prmt1 was further supported by the fact that Prmt1 is induced in endothelial cells in response to shear stress^46^ and changes in Prmt1 levels correlated with ADMA generation^26^. However, direct experimental evidence for Prmt1 overexpression in vascular diseases is still lacking. Further studies will be required to clarify the involvement of Prmt1 in ADMA-related vascular pathologies. Regardless, our current study supports a critical function of Prmt1 in vascular homeostasis. Consistently, Prmt1 expression was found to be reduced in the transcriptome datasets of aortas of patients with abdominal aortic aneurysms and aged aortas. Furthermore, Prmt1 inhibition by Fura treatment in VSMCs reduced contractile properties concurrent with the reduced contractile gene program. Thus, we believe that enhancing Prmt1 activation might be a therapeutic strategy to treat vascular diseases associated with reduced contractile properties. In conclusion, the present study demonstrates that VSMC Prmt1 is essential for vascular homeostasis and that its ablation exacerbates vascular remodeling and abnormalities. Furthermore, Prmt1 is critical for the induction of myocardin and the contractile gene program (Fig. 8h).

## Supplementary information

Supplementary Information
