# Supplementary material for: Inducible Prmt1 ablation in adult vascular smooth muscle leads to contractile dysfunction and aortic dissection
Source: Exp Mol Med. 2021 Oct 11;53(10):1569–79. doi: 10.1038/s12276-021-00684-x (PMC8568946; doi:10.1038/s12276-021-00684-x)
Supplement: Supplementary file 1 — Supplementary Information [file 12276_2021_684_MOESM1_ESM.pdf]

Supplementary Information

Inducible Prmt1 ablation in adult vascular smooth muscle leads to contractile dysfunction and aortic dissection

Jung-Hoon Pyun[1,2,#], Byeong-Yun Ahn[1,2,#], Tuan Anh Voung[3], Su Woo Kim[1,2], Yunju Jo[1], Jaehyung Jeon[4], Seung Ho Baek[4], Jaewon Kim[4], Sungsu Park[4,5], Gyu-Un Bae[6], Jun-Hyuk Choi[7], Jae-Ryong Kim[8], Dongryeol Ryu[1], Sang-Jin Lee[3*], Jong-Sun Kang[1,2*]

[1]Department of Molecular Cell Biology, [2]Single Cell Network Research Center, Sungkyunkwan University, Suwon, South Korea. [3]Research Institute of Aging Related Diseases, AniMusCure Inc., Suwon, South Korea. [4]School of Mechanical Engineering and [5]Department of Global Biomedical Engineering, Sungkyunkwan University, Suwon, South Korea. [6]College of Pharmacy, Sookmyung Women's University, Seoul, South Korea. [7]Department of Pathology, College of Medicine, Yeungnam University, [8]Department of Biochemistry and Molecular Biology, Smart-aging Convergence Research Center, Yeungnam University College of Medicine, Daegu, South Korea

***Correspondences:** Sang-Jin Lee (animus_sjlee@animuscure.com) & Jong-Sun Kang, (kangj01@skku.edu)

Supplementary Figures

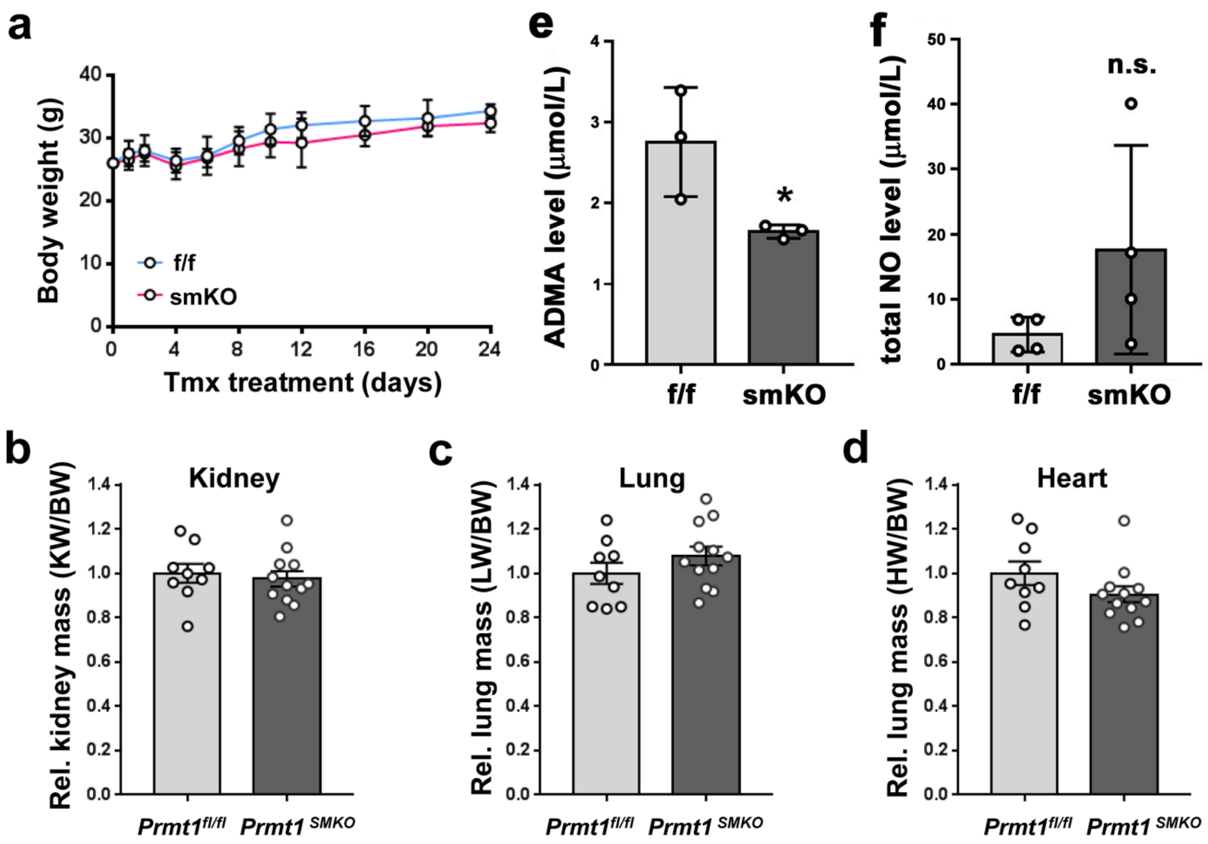

Figure S1.

Supplementary Figure 1 Inducible Prmt1 ablation in adult VSMCs does not greatly alter body and organ weights. a Body weights of f/f and smKO mice during tamoxifen treatment (n = 24). Data represent means ± SEM. *$P < 0.05$, Student's *t*-test. **b-d** Relative kidney (KW), Lung (LW) and Heart weight (HW) / Body weight (BW) of f/f and smKO mice treated with tmx for 20 weeks (n = 9-12). Data represent means ± SEM, Student's *t*-test. **e**, **f** Serum ADMA and NO level from f/f and smKO mice treated with tmx for 20 weeks (n = 3 - 4). Data represent means ± SEM, Student's *t*-test.

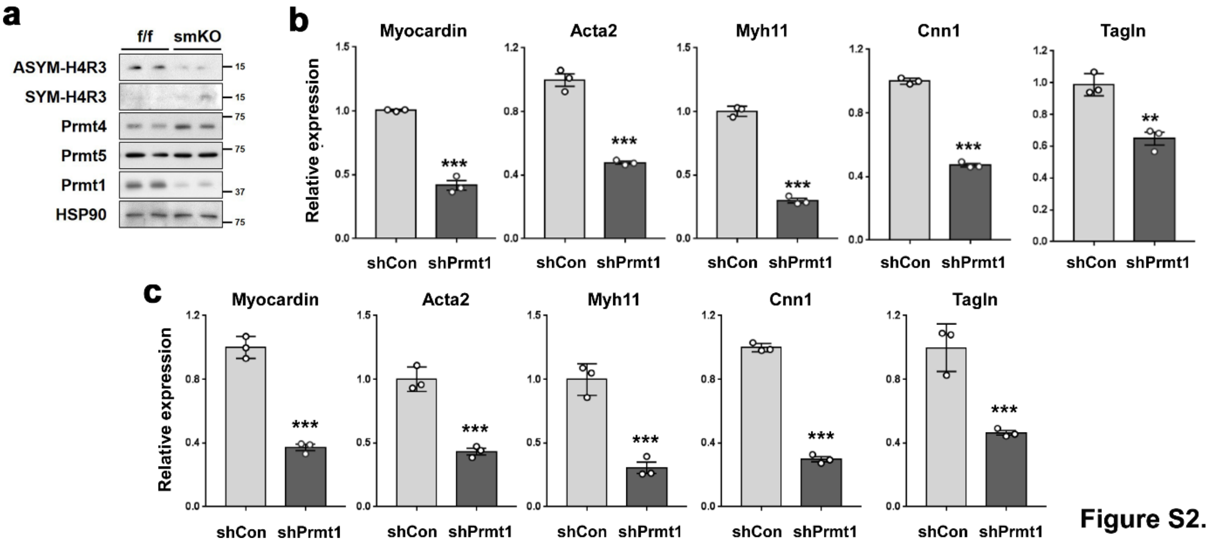

Supplementary Figure 2 Prmt1 depletion in VSMCs reduces Myocardin and its target contractile genes. a Protein analysis for Prmt1, Prmt4 and Prmt5 expressions in aorta isolated from f/f and smKO mice. **b** qRT-PCR analysis of Myocardin and its target genes in RVSMC/shCon or RVSMC/shPrmt1 cells. Data represent means ± SEM. $**P<0.01$, $***P<0.001$, Student's *t*-test. **c** RNA expression analysis for Myocardin and its target genes in Prmt1-depleted A7R5 cells. Data represent means ± SEM. $***P < 0.001$, Student's *t*-test.

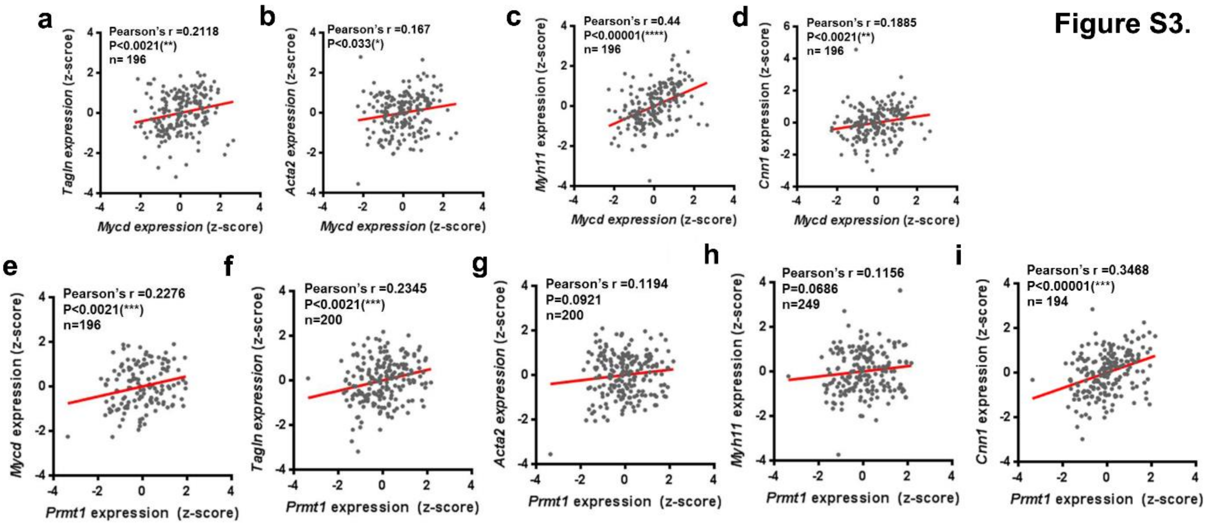

Supplementary Figure 3 A positive correlation of Prmt1, Myocardin and its target genes in VSMC transcriptomes from public database. a-d Covariation analysis of public dataset

for the expression of Myocardin and its target contractile genes. Data represent means ± SEM. *$P < 0.033$, **$P < 0.0021$, ****$P < 0.00001$. Student's *t*-test. **e** Correlation analysis for Myocardin and Prmt1 expression. Data represent means ± SEM. ***$P < 0.0021$. Student's *t*-test. **f-i** Covariation study for the expression of Prmt1 and contractile genes. Data represent means ± SEM. ***$P < 0.0021$, ****$P < 0.00001$. Student's *t*-test.

Supplementary Table 1. Antibodies used in study.

Protein	Manufacturer	Catalog number
Prmt1	Millipore	07-404
Prmt1	Abcam	ab3768
Alpha smooth muscle actin	Abcam	Ab5694
Myosin heavy chain 11	Abcam	Ab53219
Myocardin	Abcam	ab107301
Myocardin	biorbyt	Orb1794
Gapdh	Ab frontier	LF-PA0018
Histone H4 (asymmetric di methyl R3)	Abcam	ab194683
Histone H3 (Acetyl K9)	Abcam	ab4441
Histone H3 (Tri-methyl K27)	Abcam	ab6002
HSP90	Abcam	ab13495

Supplementary Table 2. Primer sequences used in study.

	Forward(5'→3')	Reverse(3'→5')
Acta2 (rat)	CACGAAACCACCTATAACAGCA	GAATATTTGCGTTCTGGAGGAG
Myosin heavy chain 11 (rat)	GAAAGGAAACACCAAGGTCAAG	CTCACTGCGAAGTTTCTTGTG
Calponin1 (rat)	ATCATTGGCCTACAGATGGGC	AGCGTGTCACAGTGTTCCAT
Tagln (rat)	AAGAATGGCGTGATTCTGAGC	CTGCCTTCAAGAATTGAGCC
Acta2 (mouse)	GCTTCGCTGGTGATGATGCTC	AGTTGGTGATGATGCCGTGTTC
Myosin heavy chain 11 (mouse)	TGCCGACACCGCCTACAGAAG	TTTCCAGCTCCCCCGTGAT
Calponin1 (mouse)	GGACCAGGCGACCATCAG	TAGGCAGAGTTGTAGTAGTTGTG
Tagln (mouse)	TGAGCCAAGCAGACTTCCAT	TGTTGAGGCAGAGAAGGCTTG
Myocardin (rat)	CAAGGGTGTGCACAGATGACT	ATGATCTTCCCTGGCATCCG
Myocardin (mouse)	AGTTTTCTGGGGACACTGGC	GCCGTAACTGTAAGACTGATCG
Gapdh (mouse)	CAGTGCCAGCCTCGTCCCGTAGA	CTGCAAATGGCAGCCCTGGTGAC
Gapdh (rat)	GACATGCCGCCTGGAGAAAC	AGCCCAGGATGCCCTTTAGT
Myocardin (chip)	GCAAAGAGTTAAGAGCCTGCT	CCAGGAGTGTCATGGTGAGTC
SRF (chip)	GCGAGTTCGGTATGTCTATGCAA	CCCAGCTTGGCTCGGTAACAT
